# Metabolic and co-expression network-based analyses associated with nitrate response in rice

**DOI:** 10.1186/1471-2164-15-1056

**Published:** 2014-12-03

**Authors:** Viktoriya Coneva, Caitlin Simopoulos, José A Casaretto, Ashraf El-kereamy, David R Guevara, Jonathan Cohn, Tong Zhu, Lining Guo, Danny C Alexander, Yong-Mei Bi, Paul D McNicholas, Steven J Rothstein

**Affiliations:** Department of Molecular and Cellular Biology, University of Guelph, Guelph, ON N1G 2W1 Canada; Department of Biology, McMaster University, Hamilton, ON L8S 4L8 Canada; Syngenta Biotechnology Inc, 3054 Cornwallis Rd, Research Triangle Park, NC 27709 USA; Metabolon Inc, 617 Davis Dr Ste 400, Durham, NC 27713 USA; Department of Mathematics and Statistics, McMaster University, Hamilton, ON L8S 4L8 Canada

**Keywords:** Co-expression network, Metabolite profiling, Nitrogen limitation, Rice, Trancriptome clusters

## Abstract

**Background:**

Understanding gene expression and metabolic re-programming that occur in response to limiting nitrogen (N) conditions in crop plants is crucial for the ongoing progress towards the development of varieties with improved nitrogen use efficiency (NUE). To unravel new details on the molecular and metabolic responses to N availability in a major food crop, we conducted analyses on a weighted gene co-expression network and metabolic profile data obtained from leaves and roots of rice plants adapted to sufficient and limiting N as well as after shifting them to limiting (reduction) and sufficient (induction) N conditions.

**Results:**

A gene co-expression network representing clusters of rice genes with similar expression patterns across four nitrogen conditions and two tissue types was generated. The resulting 18 clusters were analyzed for enrichment of significant gene ontology (GO) terms. Four clusters exhibited significant correlation with limiting and reducing nitrate treatments. Among the identified enriched GO terms, those related to nucleoside/nucleotide, purine and ATP binding, defense response, sugar/carbohydrate binding, protein kinase activities, cell-death and cell wall enzymatic activity are enriched. Although a subset of functional categories are more broadly associated with the response of rice organs to limiting N and N reduction, our analyses suggest that N reduction elicits a response distinguishable from that to adaptation to limiting N, particularly in leaves. This observation is further supported by metabolic profiling which shows that several compounds in leaves change proportionally to the nitrate level (i.e. higher in sufficient N vs. limiting N) and respond with even higher levels when the nitrate level is reduced. Notably, these compounds are directly involved in N assimilation, transport, and storage (glutamine, asparagine, glutamate and allantoin) and extend to most amino acids. Based on these data, we hypothesize that plants respond by rapidly mobilizing stored vacuolar nitrate when N deficit is perceived, and that the response likely involves phosphorylation signal cascades and transcriptional regulation.

**Conclusions:**

The co-expression network analysis and metabolic profiling performed in rice pinpoint the relevance of signal transduction components and regulation of N mobilization in response to limiting N conditions and deepen our understanding of N responses and N use in crops.

**Electronic supplementary material:**

The online version of this article (doi:10.1186/1471-2164-15-1056) contains supplementary material, which is available to authorized users.

## Background

Limiting nitrogen (N) conditions greatly affect plant growth and bring about morphological and developmental adaptations such as increased root/shoot ratio, early transition to flowering and early senescence [1]. Consequently, the application of N fertilizers has become a major input expenditure used to obtain optimal growth and high-yielding crops [2]. Nonetheless, it has been estimated that less than 40% of applied nitrogen is used by crops and most is lost through denitrification, volatilization, leaching, and runoff which in turns causes pollution to the atmosphere and aquatic environments [3]. Thus, during the last decades efforts have been directed to improve nutrient management practices and breeding for crop varieties with high nitrogen use efficiency (NUE) [4–6].

Several studies have shown genetic differences in N uptake and/or grain yield per unit of N applied in different crops including maize, wheat, rice, sorghum, and barley [7–12]. Rice represents a major food source for about half of the world’s population, and thus its production is of great importance to food security [13]. As in other major crops, rice productivity in past decades has been improved not only by breeding, but also by significantly increasing the use of N fertilizers. Several countries in Asia have attained high rice yield levels at the expense of elevated fertilizer use yet remain with relatively low NUE values [14]. This leaves opportunities for improvement through better N management procedures and development of varieties with high NUE. Increasing NUE requires a better understanding of the genetics behind N uptake, metabolism and remobilization [6, 15]. Genetic variation of N uptake, remobilization and metabolism pertaining to NUE has been reported in rice [9, 16–18]. Although functional analyses have been performed to elucidate how particular genes affect physiological aspects of rice growth and yield under N limiting conditions [19–21], the broad molecular mechanisms controlling genetic variations among different cultivars for NUE are far from being understood.

Global transcription profiling using microarrays has been a successful approach to examine molecular aspects of nutrient and stress responses. In rice, few analyses of transcriptome responses to nitrate and ammonium starvation have been performed [22–24]. However, data comparisons across studies are difficult to perform because of disparities in microarray platform and/or analysis employed and differences in growing and treatment conditions of the experiments. In addition, one of the challenges in global gene expression analysis is the large number of genes (typically thousands) and a discrete number of samples which pose problems to typical statistical interpretations. Thus, several data reduction methods have been proposed to capture the relevant information using a smaller set of variables (genes) [25]. In contrast to analyses of differential gene expression, network analyses aim to explain patterns of transcriptome organization, whereby the identification of clusters, or modules, of co-expressed genes across conditions are identified. Analysis of a network structure has the potential to yield insight into the regulation of a biological process or response, which can be hidden in direct comparisons of differential gene expression between conditions. In this work, we constructed and analyzed eigengene networks to identify transcriptome clusters associated with the response of rice plants to N availability. Furthermore, adaptation to low N has been shown to involve a complex reorganization of multiple aspects of whole-plant metabolism [22, 26–28] reflected in reduced levels of amino acids and proteins, secondary metabolites, notably anthocyanins, as well as alterations in carbohydrate metabolism reflected in changes in chlorophyll levels and starch accumulation [15, 29]. Hence, to better understand how the metabolomes of rice leaves and roots respond to N limitation, and to specifically compare the low N adapted response versus the response to a sudden reduction in N availability, we also conducted a survey of metabolic changes under sufficient and limiting N conditions providing a correlation platform with the expression responses.

## Results

### Identification of gene expression clusters associated with nitrogen limitation in leaves and roots

**Figure 1:**
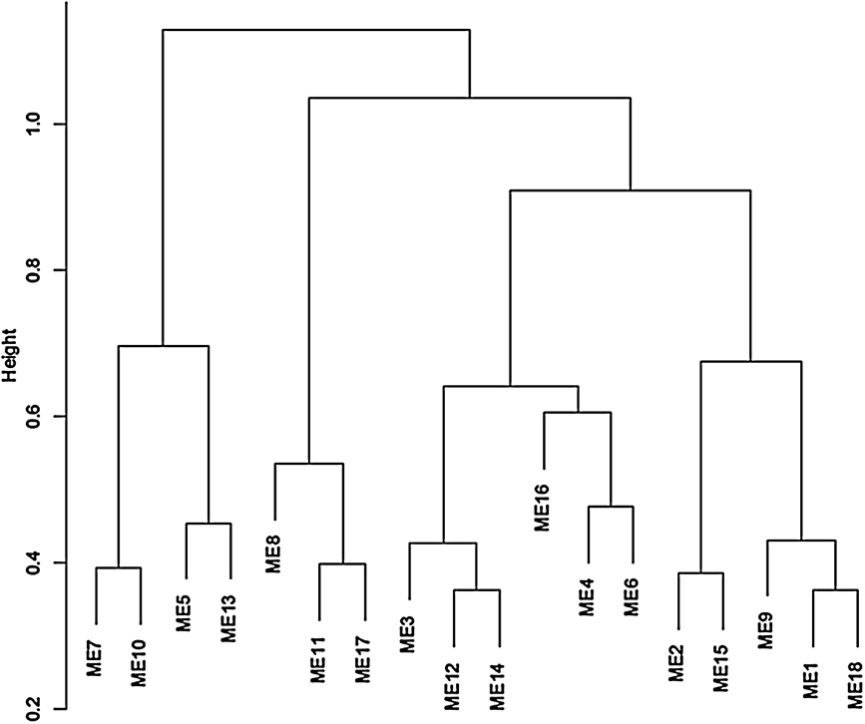
**Dendrogram of merged module eigengenes.** The dendrogram depicts the 18 clusters generated by applying a dynamic tree cutting function after hierarchical clustering. Original clusters (modules) (Additional file 1) with eigengene similarity exceeding 0.65 were merged to create the merged clusters.

**Figure 2:**
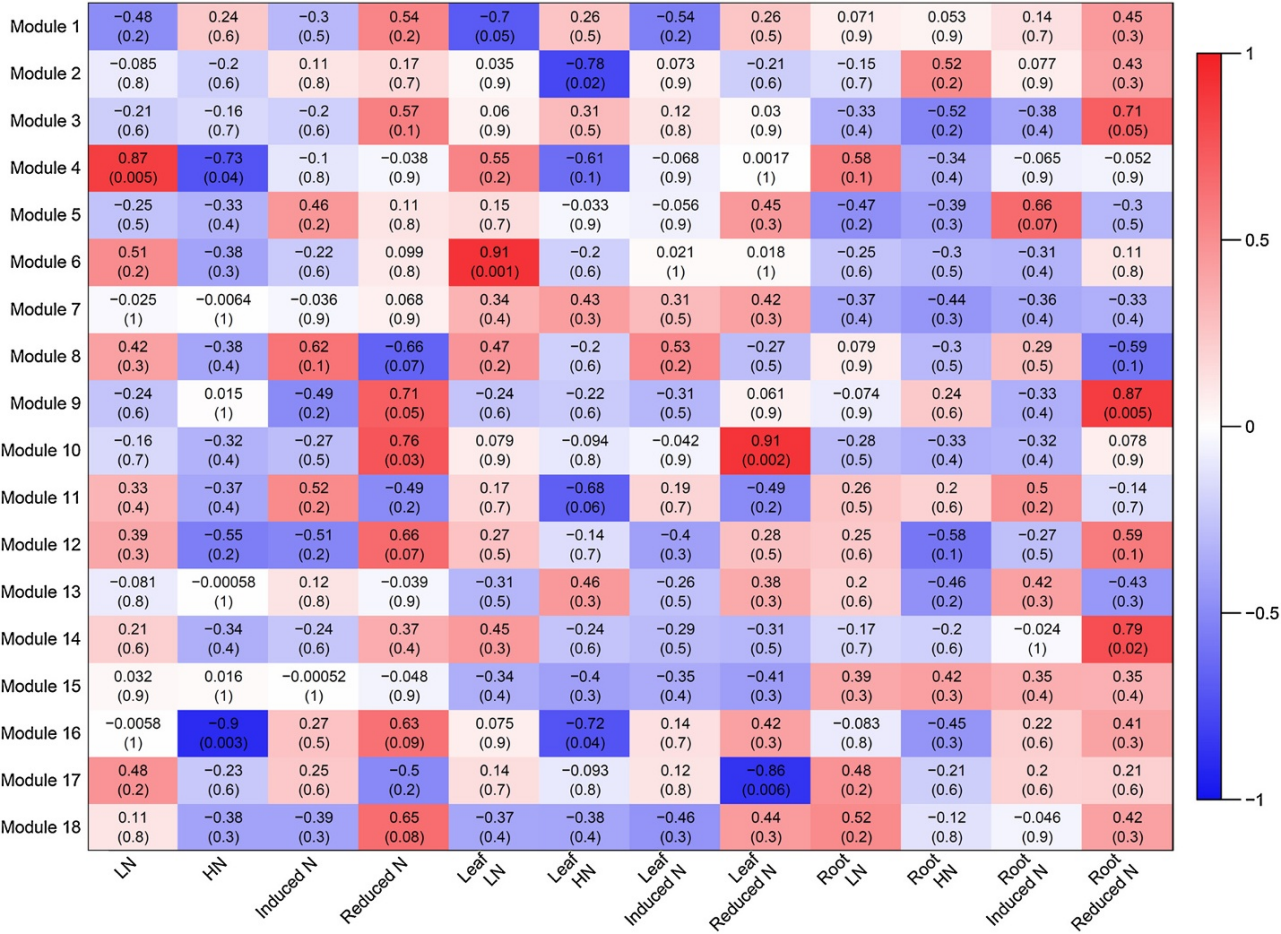
**Heatmap representing the strength and significance of correlations between module eigengenes and binary nitrogen condition/tissue combinations.** Pearson’s correlation coefficient is used as the correlation descriptor (red and blue for positive and negative correlations, respectively), and p-values are found in brackets. LN, limiting N; HN, sufficient N; Induced N (LN to HN); Reduced N (HN to LN).

Limiting and sufficient nitrogen conditions for rice grown in hydroponic and soil systems have been established previously by our group [30]. For hydroponic growth, we have determined 3 mM nitrate as sufficient N, 1 mM as mild-limiting (growth and biomass reduction start to be visible) and 0.3 mM as severe-limiting (severe symptoms are visible). In this work we used two nitrate levels, 3 mM (or HN) and 0.3 mM (or LN) representing sufficient and severe-limiting N, respectively. Rice plants were grown under sufficient (HN) and limiting (LN) N conditions or switched from HN to LN (reduction) or LN to HN (induction) as described (Methods). Total RNA was extracted from leaves and roots and used for cDNA synthesis to profile the transcriptome using microarrays. Both control probe sets and probe sets that mapped to multiple loci in the genome were removed from the analysis, reducing the rice dataset from 34,873 to 33,602 probesets. A weighted gene co-expression network was created using the WGCNA R package [31]. The resulting TOM matrix was grouped by hierarchical clustering. A total of 144 clusters (modules) of possible genetic networks were identified (Additional file 1). The large number of clusters was further reduced by merging similar clusters in order to facilitate analyses and to allow for clusters large enough to contain significant gene ontology (GO) terms (Figure 1). Each of the resulting 18 clusters was then analyzed for functional enrichment using the agriGO analysis tool (http://bioinfo.cau.edu.cn/agriGO). The results of this analysis are summarized in Table 1 and a complete list of enriched GO terms is included in Additional file 2.Eigengenes for each cluster were determined (see Methods) allowing us to evaluate the significance of a cluster to specific experimental conditions, in this case, each tissue and nitrogen condition combination. Correlations between module eigengene value, N treatment and tissue type were calculated and the results are illustrated as a heatmap (Figure 2). A first observation is that samples from roots and leaves seem to show distinct responses to N treatments. Ten out of the 18 clusters are significantly correlated (p < 0.05) to at least one condition and five of those were significantly correlated to reduced N treatments. Entities represented in these clusters could offer insight into the molecular mechanisms of adaptation to N limitation. The most significant correlations were those observed in Modules 4, 6, 9, and 10 that presented 0.87 (in LN, p < 0.005), 0.91 (LN, leaves, p < 0.001), 0.87 (reduced N, roots, p < 0.005), 0.91 (reduced N, leaves, p < 0.002), respectively (Figure 2). Interestingly, no clusters show significant correlations to N induction treatments.

**Table 1:** **Summary of the number of entities and enriched GO terms in each validated cluster**

Cluster	Entities in cluster	Number of GO terms enriched
Module 1	469	1
Module 2	376	11
Module 3	743	0
Module 4	270	9
Module 5	157	15
Module 6	2880	21
Module 7	11861	69
Module 8	694	3
Module 9	1343	4
Module 10	3337	4
Module 11	610	24
Module 12	390	1
Module 13	102	0
Module 14	604	0
Module 15	8907	214
Module 16	431	0
Module 17	195	0
Module 18	233	0

A complete list of enriched GO terms in each cluster is provided in Additional file 2.

### Functional enrichment analysis of gene clusters associated with nitrate conditions suggests tissue-specific aspects of the nitrogen adaptation and reduction responses

**Figure 3:**
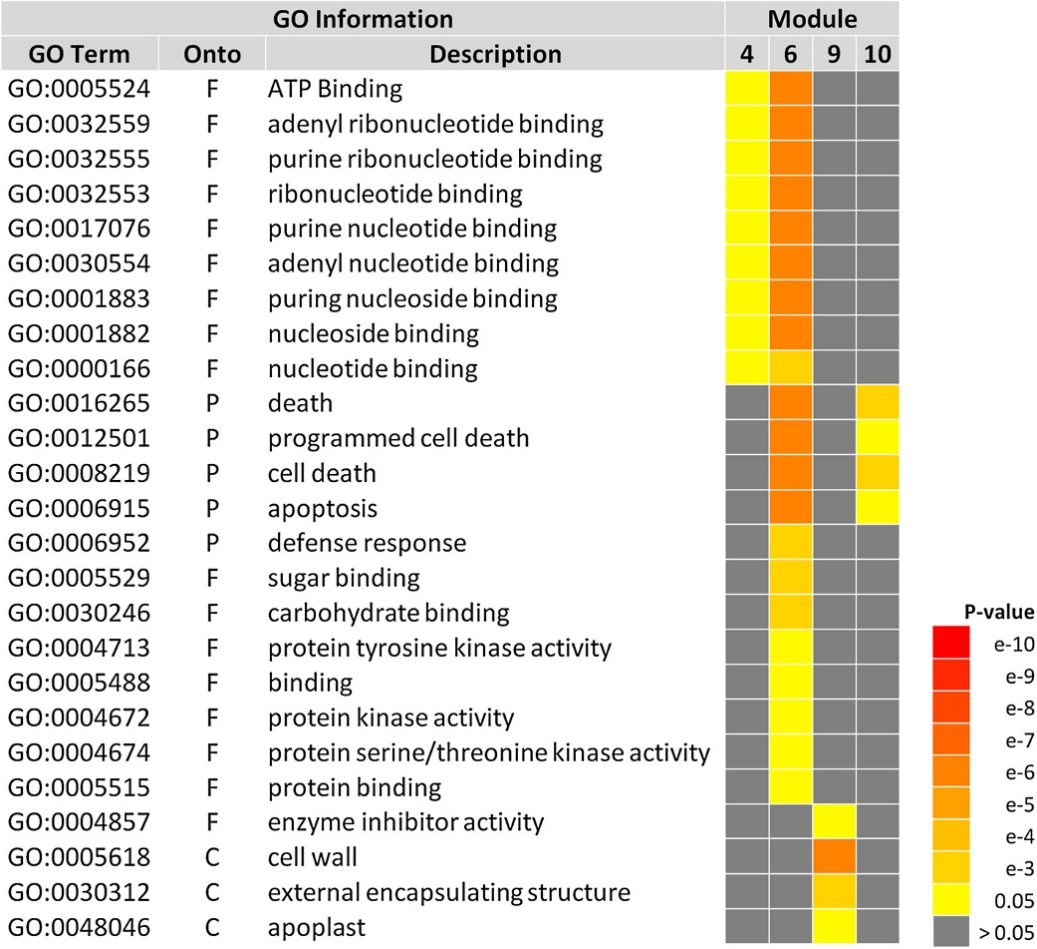
**Summary of significantly enriched GO terms in Modules 4, 6, 9, and 10.** SEA analysis was performed to determine enrichment of significant GO terms in the clusters of interest. Only significant GO terms associated with the clusters are displayed. Colored boxes indicate levels of statistical significance according to the scale (yellow to red represent decreasing p-values; and gray represents a non-significant result). Onto refers to the ontology category: F, Molecular function; P, Biological process; C, Cellular component.

Gene Ontology (GO) enrichment analysis was performed on all clusters (Additional file 2). Of particular interest are the GO enrichment terms of Modules 4, 6, 9, and 10 as these were identified to most robustly reflect tissue specific responses to N limitation (Figure 3). Modules 4 and 6 associated with the adapted LN response are enriched for molecular function terms related to nucleoside/nucleotide (GO:0001882, GO:0000166), purine (GO:0032559, GO:0033555, GO:0033553, GO:0017076, GO:0030554, GO:0001883) and ATP binding (GO:0005524). Module 4 is correlated to LN conditions in general, while Module 6 is associated with LN specifically in leaves. In addition to GO terms common to these LN-associated clusters, Module 6 also contains unique enriched terms associated with defense response processes (GO:0006952) and molecular functions related to sugar/carbohydrate binding (GO:0005529, GO:0030246), protein binding (GO:0005515) and protein kinase activities (GO:0004713, GO:0004672, GO:0004674). Interestingly, Modules 6 and 10, associated with sub-optimal N conditions in leaves show a common significant enrichment of cell-death related terms (GO:0016265, GO:0012501, GO:0008219, GO:0006915). Module 9, which is associated with the response of roots to reducing N conditions, reflects gene functions associated with enzyme activity at the cell wall and apoplast (GO:0005618, GO:0030312, GO:0048046). These findings suggest that distinct leaf and root transcriptome-level responses are utilized by rice plants to cope with limiting N conditions. Additionally, although some commonality exists in the response of rice organs to limiting and reducing N, these conditions seem to elicit distinct responses, particularly in leaves.

To substantiate our approach to transcriptome analysis, we compared the enrichment of GO terms between a list of differentially expressed genes in leaves (LN vs. HN) and entities in Module 6, associated with LN in leaves (Additional file 3). GO terms pertaining to nucleotide and purine binding/metabolism are similarly significant in both instances lending support to the notion of the biological significance of these processes in the response of rice leaves to N limitation.

### Statistical analysis of module membership suggests putative transcription factor-encoding genes as candidate regulators of the response to limiting nitrogen in rice

Nitrate initiates rapid changes in metabolism and gene expression where protein phosphorylation and transcriptional activation are involved [32]. Also, several transcription factors have been identified as potential regulators of the global gene expression response to nitrate [33, 34]. Further, the successful identification of transcriptional regulators of glucosinolate metabolism with the use of condition-specific gene expression correlation data [35] provides a proof of principle for the utility of gene network analyses to yield candidate regulators. Hence, we evaluated the centrality of transcription factor encoding genes to each of the 18 clusters in our dataset. In order to evaluate whether any putative transcription factor-encoding genes are central to the each of the clusters, a list of all putative transcription-related entities in each cluster was obtained by assigning cluster entities to MapMan bins based on their putative biological function [36]. The “regulation overview” pathway and the “Rice_japonica_mapping_merged_08” mapping were used to extract entities assigned to the bin 27 “transcription” (Additional file 4). A total of 2,103 entities were assigned to the biological function “regulation of transcription” using this approach. Next, entities within each cluster were ranked in order of decreasing module membership score. Module membership (MM) is a measure of the correlation of each entity to the eigengene describing the cluster. Thus, MM provides a quantitative measure of the importance or centrality of each entity to the cluster. Following the ranking of entities by descending MM score within each cluster, this list was queried for the highest-ranking entity with putative transcription factor annotation. Finally, we tested the significance of the ranking (see Methods). The rank of the highest ranking transcription factor annotated entity and the significance of its position is listed in Additional file 5. A similar outcome was obtained after performing rank analysis based on two other rice transcription factor-related annotation databases: PlnTFDB (http://plntfdb.bio.uni-potsdam.de/v3.0/index.php?sp_id=OSAJ) and DRTF (http://drtf.cbi.pku.edu.cn/index.php) (Additional file 5). The top-ranking transcription factor in Module 14, LOC_Os11g31330 encoding a NAC domain-containing protein, has a rank significantly higher than predicted by a random distribution (p-value = 0.0481). Module 14 is most highly correlated with reducing N conditions in roots (Figure 2). Interestingly, the next highest ranking transcription factor present in Module 11 (although less significant, p = 0.06), LOC_Os05g35170, is also a member of the NAC family of transcription factors. According to a public expression database (RiceXPro, [37]), LOC_Os11g31330 is specifically expressed during seed development, while LOC_Os05g35170 is expressed in most tissues, with highest expression in roots. These observations provide us with potential candidates for forward genetic approaches to further investigate the significance of these NAC transcription factors as regulators of the response to N limitation in rice.

### Metabolic profile of roots and leaves of rice plants subjected to limiting and sufficient nitrogen conditions

**Figure 4:**
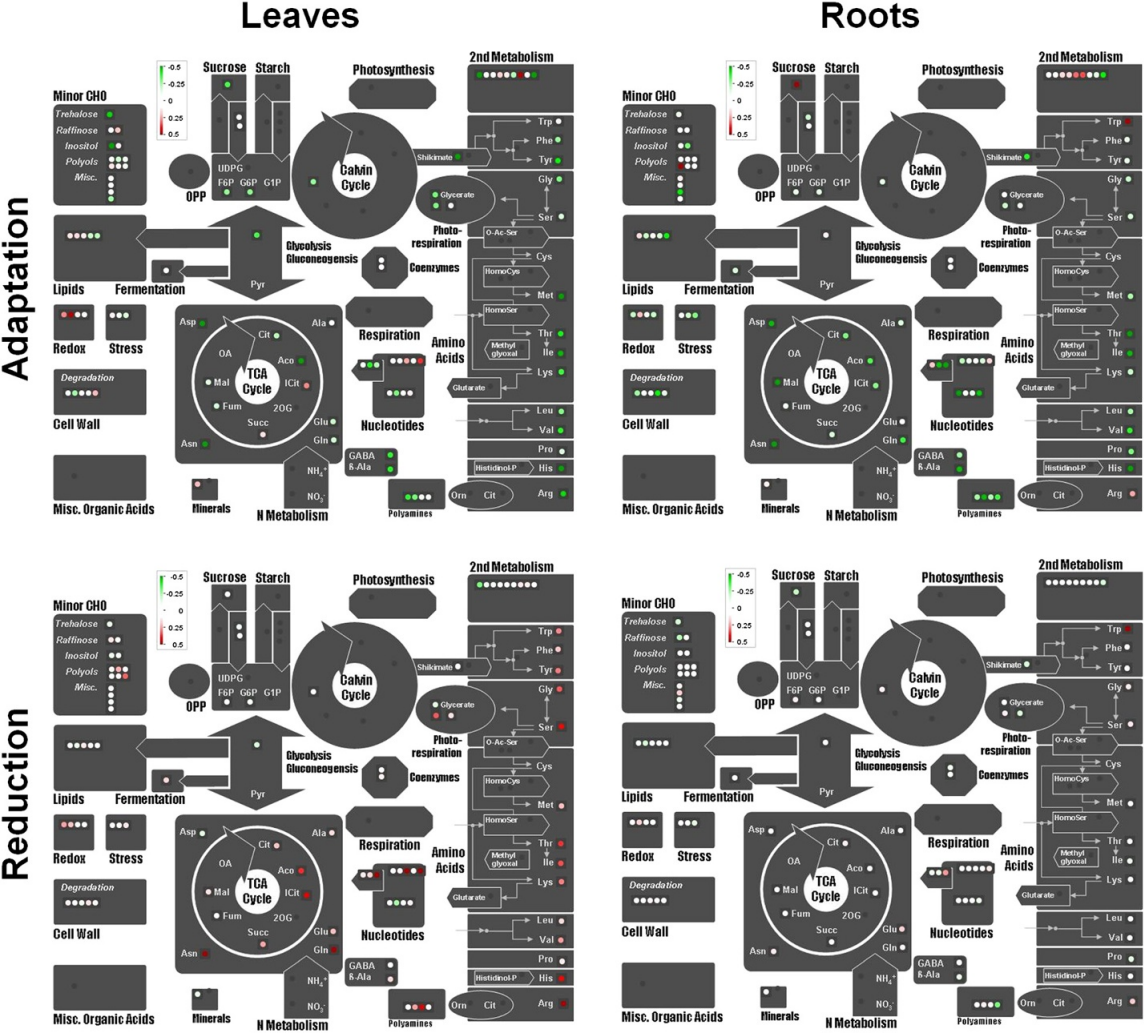
**Overview of metabolites altered in N adaptation and N reduction conditions.** Diagrams of metabolic pathways are presented as MapMan overview of metabolites altered in rice leaves and roots between pairs of conditions: sufficient nitrate (HN) vs. LN (Adaptation) and HN vs. HN to LN (Reduction). Statistically significant differences (at α = 0.05) in each comparison are represented by a false color heat map (red, increase; green, decrease).

A comprehensive metabolite profile analysis of rice samples was performed in parallel to the co-expression analysis. A total of 457 metabolites were successfully detected and 184 of these were identified using an in-house library (see Methods). We focused our analysis to address two main lines of comparison: between tissues and between the adaptation to limiting N (LN) vs. N reduction (HN to LN) treatments. To examine the adaptation to LN condition, HN and LN conditions were compared. Similarly, to obtain metabolite level changes significant to the reduction and induction conditions, shift-related changes were contrasted to plants grown under the same initial condition, i.e. (LN to HN) compared to LN for induction, and (HN to LN) compared to HN for reduction. Additional file 6 contains a summary of the number of significant metabolites in each of the categories of interest. A higher number of biochemicals are responsive to changes in N conditions in leaves compared to roots (212 or 46% of the total detected in leaves vs. 136 or 30% in roots). Second, most of the differences observed in leaves occurred in response to LN and when shifted to reducing N treatment. Interestingly, both leaves and roots exhibited a considerable non-proportional response pattern in reference to N level; that is, compounds which are reduced in the LN condition and have elevated levels upon a reduction treatment. This pattern is specific to the reduction and is not common with the induction treatment. Significant metabolite changes were mapped to metabolic pathways using MapMan (Figure 4) [36] and all identified compounds presenting significant changes in leaves and roots to different nitrate treatments are listed in Additional files 7 and 8. Most amino acids were found at reduced levels in leaves of plants grown in LN conditions, while the same tissue showed higher levels of amino acids when a sudden N limitation is imposed illustrating a non-proportional response (Figure 4; Additional file 7). One possibility is that elevated amino acid contents observed in the reduction condition may be the result of general protein degradation processes. To address this possibility, we examined our metabolome data for evidence of increased protein degradation. However, the absence of elevated levels of post-translationally modified amino acids or dipeptides in the reduction dataset indicates that protein degradation is likely not the cause of the non-proportional patterns of amino acid abundance across N conditions (Additional file 8). This suggests that reducing N conditions may be causing a rapid release and assimilation of organelle sequestered nitrate (e.g. vacuolar). Indeed, 19 of the 20 proteinogenic amino acids, as well several amino acid metabolites, showed a significant increase in terms of fold change in the reducing condition. The most notable examples in rice leaves were asparagine (7-fold), glutamine (4-fold), arginine (3-fold) and gamma-glutamylglutamine (a glutathione cycle derivative of glutamine; 5.5-fold). Interestingly, the compounds with the largest increase in reducing nitrogen conditions were asparagine and allantoin, both relevant compounds in nitrogen transport and storage (Table 2). This phenomenon was strongest in leaves followed by roots. Allantoin, a peroxisome-produced product of purine degradation, was 8 times more abundant in the reducing nitrate shift treatment, suggesting that this catabolic pathway may have a role in increasing N remobilization under N limiting conditions. In addition, significant changes were observed in the present dataset for other purine metabolites. AMP and two catabolic products of cyclic AMP (2’-AMP and 3’-AMP) increased in response to the drop in nitrate concentration. cGMP also increased after shifting from HN to LN though the change was not statistically significant. However, it accumulated more under LN conditions (Table 2). Together, the changes in all these nucleotide metabolites suggest active second messenger activity involved in nitrate regulation.

**Table 2:** N**ucleotide metabolism-related compounds under nitrate treatments**

		Leaf			Root		
Metabolite	Pathway	HN/LN	LN-HN / LN	HN-LN / HN	HN/LN	LN-HN / LN	HN-LN / HN
Allantoin	Purine metabolism, urate metabolism	1.63	1.00	8.17*	7.68*	1.33	1.81
3'-AMP	Purine metabolism, adenine containing	0.67	0.71	2.21*	1.00	1.00	1.01
AMP		0.57	1.46	6.16*	1.04	1.00	0.96
2'-AMP		1.02	1.13	2.04*	3.44*	1.36	0.45*
2',3'-cGMP	Purine metabolism, guanine containing	0.42*	0.83	1.56	1.29	0.54	1.04

Metabolic profile of compounds associated to the nucleotide metabolism super pathway that varied in leaves and roots of rice plants subjected to different nitrate treatments. Fold change (ratio of means) are shown. Asterisks indicate significant change for the indicated *t*-test (p<0.05).

## Discussion

### Co-expression network analysis reveals enrichment of functions essential for nitrate signaling

Differential gene expression surveys using microarray technology on N deficiency stress response have been reported for rice and other crops [22–24, 38]. However, differential expression analyses usually ignore the correlations that may exist between gene expression profiles. This makes it difficult to prioritize functions or to uncover the underlying regulatory mechanisms. In contrast, in the present expression network analysis, we hypothesized that gene expression profiles in response to N availability can be highly correlated and can thus be grouped into gene clusters or co-expression clusters. We have taken advantage of gene co-expression clusters to analyze rice responses to N adaptation, N induction and N reduction treatments and to gain insights on the regulation of plant responses to this nutrient stress at the molecular, metabolic and physiological levels. In such clusters, the module eigengene –a mathematical descriptor of the cluster– was used to summarize the expression profile of each cluster [39]. Furthermore, in this work, metabolic profile analyses were included to further explore rice responses to nitrate changes.

Our network analysis organized the rice transcriptome into 18 clusters containing genes with highly similar expression patterns under our set of conditions. Further, we calculated the association of each cluster with N treatments and tissue type (Figure 2). Using GO term enrichment analysis, we found terms in the clusters that presented significant correlation with whole plant LN conditions (Module 4), LN conditions in leaves (Module 6), and N reduction in leaves (Module 10) and roots (Module 9). Significant GO terms in these clusters include: nucleotide/nucleoside, purine and ATP binding; defense response processes, sugar and carbohydrate binding, protein binding, protein kinase activities, cell-death related processes and enzyme activities at the cell wall and apoplast (Figure 3). Interestingly, two of the clusters correlated with adaptation to LN presented enrichment of molecular functions associated with binding to nucleotides, purines and ATP. These terms comprise a wide spectrum of functions and include genes encoding proteins that use ATP or GTP in enzymatic activities, transport or signaling, among others. A close examination of the annotated genes revealed that most entities encode ATPases, protein kinases and receptor kinases (e.g. LRR kinases). Few others include genes for DNA and RNA helicases, GTPases, nucleotide transporters and a nucleoside kinase (Additional file 2). These functions emphasize the importance of signaling processes in response to nitrate. In a similar study, Beatty et al. [40] compared the transcriptome changes between a wild type rice genotype with a transgenic high NUE genotype after 10 and 26 days at three ammonia concentrations. Although no N induction or reduction treatments were included, the investigators found that under limiting N conditions, several induced genes in the high NUE genotype were involved in regulation of transcription and protein phosphorylation biological processes.

Phosphorylation is a ubiquitous mechanism in the regulation of pathways controlling diverse processes in plants. In the case of N related processes, for example, two calcineurin B-like-interacting Ser/Thr protein kinases, CIPK8 and CIPK23, regulate the expression of nitrate responsive genes, including nitrate transporter encoding genes and genes required for N assimilation, and affect signaling activity when N availability drops [41, 42]. In maize leaves, more than 100 phosphorylated proteins have been analyzed, including those involved in C and N metabolism, RNA helicases, and transcription and translation factors. Among them are (NADH)-nitrate reductase and proteins associated with photosynthesis [43], suggesting tight control of these metabolic routes. In Arabidopsis, rapid responses to nitrate resupply (induction) also involve changes in the phosphorylation level of proteins with signaling functions (receptor kinases), transcription factors and transporters [44].

Roles for ATP in modulating different aspects of N metabolism have been reported. Nitrate assimilation depends on the availability of ATP and reducing power supply such as NADPH and NADH [6]. In Arabidopsis cells, storage of nitrate within the vacuole is primarily mediated by the nitrate/H+ exchanger AtCLCa. It has been described that AtCLCa activity can be negatively regulated by cytosolic ATP levels, inhibiting nitrate influx into the vacuole [45]. AMP is known to prevent this inhibition [45]. Hence, physiological level of ATP is a regulatory point for nitrate use within the cell. The expression of seven genes encoding different ATPase isoforms is also up-regulated by N deficiency and N induction in rice shoots and roots. In addition, increased plasma membrane proton pump ATPase activity results in increased net uptake of nitrate and ammonia [46]. In this sense, the fact that two clusters of our dataset presented several entities associated with ATP binding and ATPase activity suggest that ATP-mediated processes have an important role in responses to N deficiency in rice.

Transcription factors are also important downstream integrators of signaling pathways and control gene expression to generate responses to nutrient limitation [34]. A significant number of genes annotated as having transcription factor activity have been identified as responsive to N treatments in rice [22, 23] and other species [33, 34, 38, 47]. We identified over 2,000 entities associated with regulation of transcription in our dataset and performed a module membership rank analysis to determine whether some transcription factors may be representatives of each cluster eigengene and thus possible regulators of the members in their own cluster. We found two NAC transcription factors that are highly ranked, one in Module 14 associated with nitrate reduction treatment in roots and another in Module 11 (Additional file 5). Potential significant roles of members of this transcription factor family in nitrate responses in plants have been documented. In an analysis of 27 Arabidopsis array data sets, ca. 10% (219/2286) of the genes that consistently respond to nitrate in roots correspond to transcription factors, and of those the third most represented family was the NACs group [34]. Additionally, Peng et al. [47] reported five NAC/NAM transcription factor encoding genes that are up-regulated by nitrate in wild type Arabidopsis plants and nine in the *nitrogen limitation adaptation* (*nla*) mutant. Other examples of N-responsive NAC transcription factors include NAC4, a key regulator of a nitrate-responsive network reflected in Arabidopsis lateral root growth in response to nitrate [48] and PtaNAC1, found to be a central regulator of root response to low N in genetic network analysis of poplar [49]. In wheat, a NAC factor has been identified for its involvement in the N mobilization process during grain development. Wheat plants with reduced *TsNAC-B1* expression display delayed senescence and 30% less protein accumulation in seeds [50]. Therefore, NAC transcription factors seem to play a role not only in rapid responses to N limitation but also in N remobilization including during the leaf senescence process [51]. Similarly, nutrient remobilization in crops is related to degradation of macromolecules and salvage of nutrients from senescing tissues. This process may occur through autophagy and related cell death events [52]. Detection of GO terms associated with cell death and apoptosis in Module 6 which is associated with LN in leaves is consistent with this observation. Further, Modules 6 and 10 (Figure 3), associated with N limitation in leaves share enrichment for cell death related terms suggesting that this may be a leaf-specific response to sub-optimal N conditions. The Arabidopsis *nla* mutant has a decreased capacity to adapt to limiting N and undergoes accelerated leaf senescence in response to these conditions [47].

### Metabolic profiling indicates rapid response for nutrient allocation under N reduced conditions

The transcriptome analysis of this work suggests that N limitation results in major reorganization of plant metabolism in a tissue and N condition specific manner. Metabolite analysis supports the observations of the transcriptome data. Not only are the responses of leaves and roots to sub-optimal N distinct, but so are the responses of each organ to growth at limiting N and reducing N treatments. A higher number of metabolite variations were detected in leaves compared to roots during short-term response to N availability. The metabolic profile suggests that rice plants under HN were more anabolically active (i.e. higher content of amino acids, hexose phosphates, sucrose, pentose phosphate pathway intermediates, etc.) compared to those plants under LN. Increased sucrose levels in response to HN suggests that leaves were operating more strongly as source tissues under the HN condition, providing carbon and energy for growth activities (protein synthesis, cell wall production, and other functions). Some nitrogen-containing compounds changed proportionally to the nitrate level (i.e. higher in HN vs. LN). For example, glutamine, asparagine, glutamate, aspartate and arginine were all either directly proportional to nitrate, or were not statistically different. Glutamine and aspartate were also directly proportional to nitrate in roots. Asparagine showed an especially strong difference in leaves and roots. Interestingly, alanine, which is derived by the glutamate-mediated transamination of pyruvate, also fell into this group, resembling the behavior of aspartate, glutamate and glutamine in both leaves and roots. Alanine may be involved in N balance in plants, as it can serve as a storage compound under certain stresses [53]. It has been reported that a barley alanine aminotransferase expressed in roots exhibits improved NUE under reduced N conditions [53]. However, it was interesting to observe, from a physiological perspective, that some compounds in leaves behaved non-proportionally with respect to N condition; that is, their levels were lower in plants grown at limiting N but elevated sharply in plants shifted from sufficient N to limiting N. It may be important that the compounds which showed the strongest increase, in terms of fold change to the reducing condition, were those directly relevant to N metabolism such as asparagine, glutamine, arginine and allantoin. Evidence for early N remobilization in shoots to support root growth has been described in mature Arabidopsis plants subjected to N starvation. When undergoing long term N stress, such plants exhibit an increase in N remobilization enzyme activities in shoots; though a larger capacity of high-affinity nitrate uptake in roots was also detected [54]. Few possibilities can explain why so many N-rich compounds (amino acids in general) are dramatically increased as rice plants were moved from sufficient nitrate to a limiting nitrate condition: (1) a rapid increase in proteolysis that might be associated with a senescence response; (2) induction of a high affinity nitrate system, possibly triggering the more rapid assimilation of residual nitrate in the plant tissues; or (3) a rapid release of sequestered nitrate, presumably from vacuolar stores [52]. Evidence for proteolysis was rather weak. A post-translationally modified amino acid form (N6-acetyllysine) which can be a marker for proteolysis as well as several dipeptides were detected, but their response pattern did not match the general amino acid response (Additional file 8). The second alternative, that is a dramatic change in the dynamics of nitrate transport by a rapid induction of a high affinity system, also seems unlikely. Since most of the induced transport and assimilation systems of this type described in the literature would involve gene induction, translation, and then transport to the leaves to allow assimilation and enzymatic alteration of many metabolite pools, this seems intuitively less plausible for a short-term response than a more direct regulatory mechanism (e.g. kinase/phosphatase cascades). Also, the expression profile of high affinity transporters represented in the array does not support this scenario. The third possibility therefore seems most likely, as it would involve protein-level mechanisms that modify transport across the tonoplast to release sequestered nitrate. This is plausible in leaves and roots if nitrate were pre-stored in both tissues and if a nitrate sensing signal were rapidly transmitted. The rate of vacuolar nitrate release has been reported in individual barley root cells, and a significant drop in vacuolar nitrate was observed in few hours [52]. Interestingly, in those experiments the nitrate released into the cytoplasm was rapidly assimilated into other compounds consistent with the metabolite profiles of the rice plants in the present study. A rapid release of nitrate upon a reduction in available N is also consistent with the elevated levels of assimilatory amino acids (asparagine, glutamine, arginine) observed here. One might also expect to see a concomitant decrease in the organic acids supplying the carbon backbones for the newly formed amino acids, as it was the case for pyruvate (for the alanine backbone; Additional file 8).

In general, one can also infer that the leaves experience a net movement of carbon compounds into secondary pathways under conditions of limiting N. Some of these compounds reversed their levels rapidly during the nitrate shift experiments. Two compounds associated with anabolic processes were glycerol-3-phosphate (G-3-P, which presented induction patterns similar to those of amino acids and sugars described above) and ferulate (maintained higher levels in roots in the LN condition). G-3-P is essential in the synthesis of membrane phospholipids, while ferulate is an important phenylpropanoid precursor in cell wall synthesis. In this sense, it was interesting to observe that one of the clusters (Module 9) associated with the root responses to HN to LN shift condition included genes that correspond to cell wall-related GO terms (including “apoplast” and “external encapsulated structure”). It is intriguing to speculate that this may reflect alterations to cell physiology in roots that affect changes in permeability to water and nutrients.

Another interesting finding from the metabolite data is the higher content of several purine metabolism compounds, specifically in reducing N conditions (Table 2). As previously mentioned, enrichment of GO terms relating to purine metabolism was observed in Modules 4 and 6, and Module 6 is highly correlated with limiting N conditions in leaves (Figure 3, Additional file 3). Allantoin, a peroxisome-produced product of purine degradation is 8 times more abundant in leaves of plants subjected to reducing N conditions. The significance of this finding could be several-fold. Accumulation of allantoin could indicate an increase in purine ring degradation, a pathway that has been shown to result in increased N recycling in source tissues for remobilization (reviewed in [55]). Particularly, N-fixing legumes utilize ureides for root to shoot N transport [56, 57]. In addition, allantoin and its product allantoate are likely involved in protecting plants during abiotic stress by quenching of reactive oxygen species (ROS) [58–60]. Reports of the protective properties of ureide compounds in response to nutrient stress exist to date [59]. Interestingly, a key enzyme in the purine degradation pathway, allantoin synthase, has been implicated as a substrate for the LRR receptor kinase Brassinosteroid Insensitive 1 [61], providing a conceptual link between purine catabolism and a phosphorylation signaling pathway regulating plant growth.

In addition, cyclic nucleotides are considered important signaling molecules and may also be relevant for nitrate (short) responses. cGMP has been suggested to play important roles in plant development and responses to stresses. Hormones such as abscisic acid (ABA), auxin (IAA), and jasmonic acid (JA) have a significant effect on cytoplasmic cGMP levels which in turn alter downstream cascade of events such as the phosphorylation status of other proteins [62]. cGMP has also been reported to be involved in signaling pathways related to nitric oxide production especially in the induction of program cell death [63], and there has been considerable research in plants related to cAMP [64]. In the present dataset we observed that cGMP, and two catabolic products of cAMP (2’-AMP, 3’-AMP) all rise in response to the drop in nitrate concentration in rice leaves. Together, the changes in these cyclic nucleotide metabolites suggest active second messenger activity involved in nitrate regulation.

### Limitations and challenges of network analysis

Whereas co-expression networks with biological relevance were identified, the high computational requirement of this analysis was a major limitation. Access to a computer with high RAM capacity (e.g. 72 GB) was needed, and such resources are not readily available to most researchers. The developers of the WCGNA package have identified this pitfall and have developed a function that allows users to complete an analysis on a standard computer by pre-clustering genes into "blocks" using a modified k-means method [65]. After blocks of similar genes are identified, TOM matrices for each block are identified in each individual TOM by average linkage hierarchical clustering. The dendrograms are cut with the dynamic hybrid tree cutting algorithm. After processing the clusters using several steps to ensure high module membership, similar clusters across all TOM matrices are merged. Previous research has found biologically meaningful genetic networks in a variety of settings using the block-wise WGCNA method [66, 67]. Although the block-wise method accommodates for a smaller amount of required RAM, a network analysis would ideally be completed on an entire data set, as pre-clustering the data could lead to artificial gene expression clusters. For this reason, an R package that can complete a WGCNA analysis with a smaller memory usage is currently in development.

## Conclusions

As a complementary tool to differential expression analysis, co-expression network analysis offers the advantage to capture relevant transcriptomic information using gene clusters. A set of clusters of co-expressed genes associated with the response of rice plants to different N conditions was identified to provide insights into biological process and regulation of N responses in crops. Incorporating some of these genes in targeted functional studies would complement and validate their implication in this process. Examination of function annotations in gene clusters with significant correlation with nitrate treatments indicated the importance of signaling transduction, transport, metabolic regulation and cell death-related processes in response to nitrate. Metabolic profiling supports the observation that N reduction elicits a response distinguishable from that to limiting N adaptation, particularly in leaves. Our data suggest that plants rapidly respond to N limitation most probably by remobilizing stored nitrate, and likely this response involves phosphorylation signal cascades and transcriptional regulation. Developing similar type of analysis to integrate responses to multiple conditions and in diverse genetic backgrounds will be valuable for expanding our understanding of nutrient use efficiency in crops.

## Methods

### Plant growth and sample collection

Seeds of rice (*Oryza sativa* ssp. japonica) cultivar Kaybonnet were germinated in Turface (calcined clay grains) for 5 days before moving to the hydroponics system. Plants were grown in growth chambers with a 16 h light cycle (6 am to 10 pm), at 29°C during the day and 23°C during the night and constant relative humidity of 70% in a hydroponic system for four weeks in sufficient (3 mM KNO_3_) or limiting (0.3 mM KNO_3_) nitrogen conditions. The nutrient solution also contained 4 mM MgSO_4_, 5 mM KCl, 5 mM CaCl_2_, 1 mM KH_2_PO_4_, 0.1 mM Fe–ethylenediaminetetraacetic acid (EDTA), 0.5 mM MES (pH 6.0), 9 mM MnSO_4_, 0.7 mM ZnSO_4_, 0.3 mM CuSO_4_, 46 mM NaB_4_O_7_ and 0.2 mM Na_2_MoO_4_ as defined in previous work [20, 30]. Twenty four seedlings were planted in a 35-L container with 25 L of the nutrient solution. pH and nitrate levels were monitored weekly as described elsewhere [26]. Fresh nutrient solution was added weekly and the pH (5.5) adjusted with phosphoric acid. After four weeks, plants grown in limiting N (LN) conditions were transferred to a sufficient N (HN) condition (induction treatment) and those grown at sufficient N conditions moved to a limiting N system (reduction). Despite differences in growth or tiller number between plants under different N treatments, all plants were at the active tillering stage. Samples (three biological replicates) of leaves and roots were taken separately at 0 h (11 am; normal and low N conditions) and 2 h after induction and reduction treatments (1 pm) and frozen immediately and stored at -80°C until further analysis.

### Microarray experiment and analysis

Five micrograms of total RNA from each sample were isolated as described [20] and used to synthesize double-stranded cDNAs. Labeling and hybridization to a Syngenta custom designed rice whole-genome Affymetrix GeneChip™ array was conducted as described in Bi et al. [20]. Array images were acquired with the GeneChip scanner 3000 and the hybridization signals were quantified using the GCOS software (Affymetrix, Inc.). A custom chip description file was created to better map the probes on the array to the gene model of rice genome (MSU Rice Genome Annotation Project release 6.1). Data from the CEL files generated were condensed to probe set level approximating genes and transcripts using this custom file with robust multichip analysis (RMA) using an implementation of the algorithm in the Refiner Array tool in the Expressionist software suite from Genedata (Basel, Switzerland). A threshold was applied to the normalized condensed data so that a probe set was excluded if it did not have a detectable signal in at least one experiment (3 replicates). A detectable signal was defined as 2 standard deviations above average background signal. Background signal was defined by unutilized spike in controls. Data have been submitted to the Gene Expression Omnibus repository (accession number GSE61370).

### Cluster identification

Transcriptomic data was pre-processed via Robust Multichip Average (RMA) default settings [68] discarding control probe sets and probe sets that mapped to multiple loci in the genome. The median gene expression values from each triplicate were used for network analysis. Gene expression clusters were identified after weighted gene expression network creation using the WGCNA package in R [31]. Firstly, a weighted adjacency matrix was calculated. This non-binary matrix described pair wise similarities between probe pairs and is calculated as follows [31]:


where *cor(x*_*i*_*x*_*j*_*)* represents the Pearson’s Correlation Coefficient between genes *i* and *j*. A soft-threshold *β* was chosen by considering both the R^2^ coefficient value of the scale-free topology fit model and the mean connectivity of the network. A decrease in mean connectivity was seen as R^2^ increases (Additional file 9). In this case a soft threshold *β* of 4 was chosen as it maintained a high mean connectivity and was the lowest observed *β* before the scale free topology curve reached R^2^ = 0.90 (Additional file 9) [63]. The resulting signed matrix included elements ranging from -1 to 1. From this, the topological overlap measure (TOM) matrix was calculated. This matrix is a measure describing how closely related probes in a probe pair are to each other relative to all other probes [69]. In other words, a pair of genes would have high topological overlap if they share a set of the same neighbors. The TOM matrix is calculated as [69]:



where *k*_*i*_ = *Σ*_*u*_*a*_*iu*_.

Hierarchical clustering with complete linkage and Euclidean distance was used to cluster the data using a dissimilarity matrix of the TOM matrix. This was calculated as:


A dynamic tree cut function from the WGCNA R package was used to identify clusters [31, 70]. The dynamic tree-cutting step was repeated five times with varying random start numbers using the set.seed() function to ensure validity of the clusters [71]. Eigengenes for each cluster were also calculated. As the first principal component of each cluster, an eigengene denotes a mathematical descriptor of each cluster (module) which allows for computations of similarity among clusters and between a cluster and any experimental condition [39]. This allowed for the correlation between module eigengenes to be calculated. From this, clusters with eigengene similarities greater than 0.65 were merged.

### Functional annotation and enrichment analysis

Cluster gene lists were exported to the agriGO Gene Onotology (GO) term enrichment analysis tool (http://bioinfo.cau.edu.cn/agriGO) [72]. Significant GO terms were identified by Singular Enrichment Analysis (SEA) using cluster Locus IDs and publically available GO term annotation from the Rice TIGR Genome Reference. Fisher and FDR statistical test methods were used with a 0.05 significance level.

### Module membership scores

Module membership (MM) scores were used to describe the relationship between gene expression values of a cluster and cluster eigengene. MM scores were calculated as [73]:


where *x* refers to the *i*-th gene of the cluster, and *E* to the eigengene of cluster *q*. For the examination of genes encoding transcription factors, entities were ranked within each cluster in order of decreasing module membership score. This ranked list was queried for the highest-ranking entity with putative transcription factor annotation. To ascertain whether the rank of each of the highest ranking putative transcription factor entity was statistically significant, 100,000 random rankings of the entities in each cluster were generated. The rank of the highest TF annotated entity was recorded. The resulting rank distributions were used in a Wilcoxon signed rank test at a significance level of 0.05.

### Metabolic profile and data analysis

Sample preparation and analysis was carried out by Metabolon Inc. (Durham, NC, USA). All samples were maintained at -80°C until processed with the automated MicroLab STAR® system from Hamilton Co. (Reno, NV, USA) and Metabolon’s proprietary series of organic and aqueous extractions. The resulting extract was divided into two fractions; one for analysis by LC and one for analysis by GC. Liquid chromatography/Mass Spectrometry (LC/MS) portion of the platform was based on a Waters ACQUITY UPLC and a Thermo-Finnigan LTQ mass spectrometer, which consisted of an electrospray ionization (ESI) source and linear ion-trap (LIT) mass analyzer. Each sample extract was split into two aliquots, dried, then reconstituted in acidic or basic LC-compatible solvents, each of which contained 11 or more injection standards at fixed concentrations. One aliquot was analyzed using acidic positive ion optimized conditions and the other using basic negative ion optimized conditions in two independent injections using separate dedicated columns. The samples destined for GC/MS analysis were re-dried under vacuum desiccation before derivatization under dried nitrogen using bistrimethyl-silyl-triflouroacetamide (BSTFA). The GC column was 5% phenyl and the temperature ramp is from 40° to 300°C in a 16 minute period. Samples were analyzed on a Thermo-Finnigan Trace DSQ fast-scanning single-quadrupole mass spectrometer using electron impact ionization. Raw mass spec data were extracted and loaded into a relational database. Compounds were identified by comparison to library entries of purified standards or recurrent unknown entities. Identification of known chemical entities was based on comparison to Metabolon’s library entries of purified standards that includes more than 2000 commercially available purified standard compounds. Biochemical data were analyzed by Welch’s two-sample t-tests to test that the means of two independent groups are equal. The relatively conservative criteria of statistical cut-offs of p≤0.05 (probability of obtaining a result as or more extreme than the observed data) and q≤0.10 (result expected to yield a false discovery rate of no more than 10%) are routinely used in metabolomic studies. For all analyses, missing values (if any) were imputed with the observed minimum for that particular compound. The statistical analyses were performed on (natural) log-transformed data to account for increases in data variance that occurs as the level of response is increased. For this study, *t*-test comparisons were performed between the means of each biochemical across the experimental groups: (1) limiting N (LN); (2) sufficient N (HN); (3) LN to HN (induction); and (4) HN to LN (reduction).

### Availability of supporting data

The datasets supporting the results of this article are available in the Gene Expression Omnibus repository (accession number GSE61370 in http://www.ncbi.nlm.nih.gov/geo/).

## Electronic supplementary material

Additional file 1: **Dendrogram of original module eigengenes.** (PDF 9 KB)

Additional file 2: **List of entities in each cluster and GO terms identified in each cluster.** (XLSX 358 KB)

Additional file 3: **Comparison of GO terms enrichment between a list of differentially expressed genes in leaves and entities in Module 6.** (PDF 32 KB)

Additional file 4: **List of all entities with the biological function “regulation of transcription”.** (XLSX 57 KB)

Additional file 5: **Module Membership (MM) ranking of transcription factor related genes within clusters.** (PDF 37 KB)

Additional file 6: **Summary of the number of changes in the matabolic profile in rice under different nitrate treatments according to Welch’s two sample** ***t*** **-test comparisons.** (PDF 37 KB)

Additional file 7: **Metabolic profile of amino acids in leaves and roots of rice plants subjected to different nitrate treatments.** (PDF 43 KB)

Additional file 8: **Metabolic profile of other identified compounds that presented significant changes in leaves and roots of rice plants subjected to different nitrate treatments.** (PDF 52 KB)

Additional file 9: **Weighted adjacency matrix that describes pair wise similarities between probe pairs.** (PDF 11 KB)
