# Supplementary material for: Metabolic and co-expression network-based analyses associated with nitrate response in rice
Source: BMC Genomics. 2014 Dec 3;15(1):1056. doi: 10.1186/1471-2164-15-1056 (PMC4301927; doi:10.1186/1471-2164-15-1056)
Supplement: Supplementary file 1 — Additional file 1: Dendrogram of original module eigengenes. (PDF 9 KB) [file 12864_2014_6767_MOESM1_ESM.pdf]

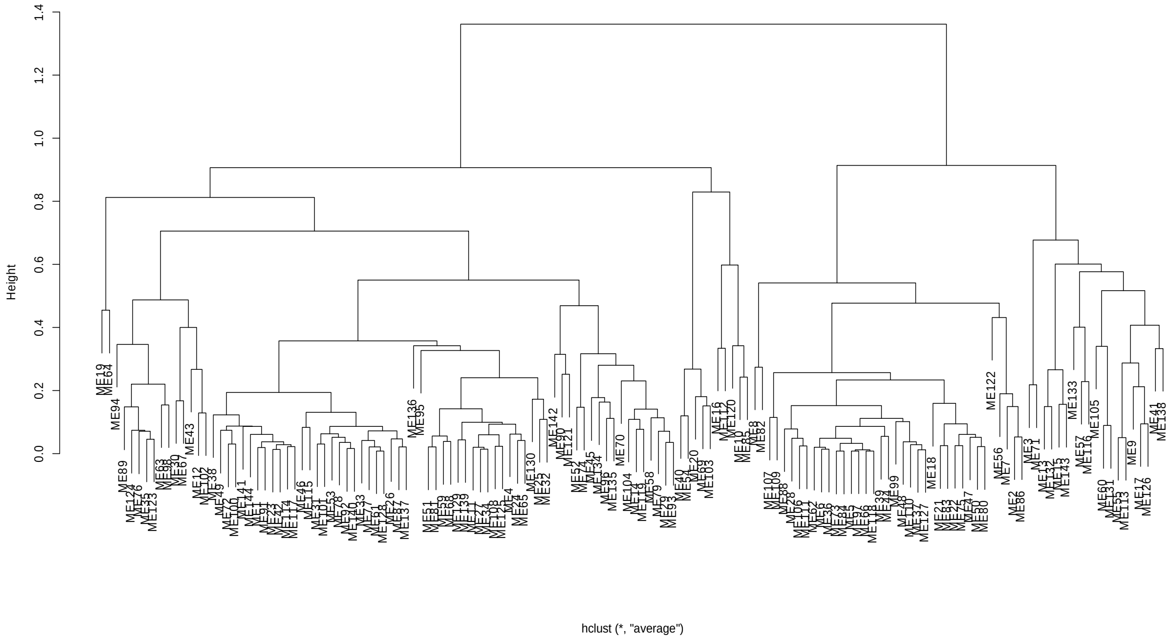

Additional file 1. **Dendrogram of original module eigengenes.** The dendogram was created after hierarchical clustering of the TOM matrix by complete linkage and Euclidean distance before merging. The tree was cut using a dynamic tree cutting algorithm. A large number of modules impeded further analysis as the modules were very similar, and did not contain enough entities for GO term enrichment analysis.
