# Supplementary material for: Metabolic and co-expression network-based analyses associated with nitrate response in rice
Source: BMC Genomics. 2014 Dec 3;15(1):1056. doi: 10.1186/1471-2164-15-1056 (PMC4301927; doi:10.1186/1471-2164-15-1056)
Supplement: Supplementary file 3 — Additional file 3: Comparison of GO terms enrichment between a list of differentially expressed genes in leaves and entities in Module 6. (PDF 32 KB) [file 12864_2014_6767_MOESM3_ESM.pdf]

GO Information			Leaf	Module 6
GO Term	Onto	Description	HN vs LN	(Leaf LN)
GO:0030554	F	adenyl nucleotide binding		
GO:0001883	F	purine nucleoside binding		
GO:0001882	F	nucleoside binding		
GO:0017076	F	purine nucleotide binding		
GO:0032559	F	adenyl ribonucleotide binding		
GO:0005524	F	ATP binding		
GO:0032553	F	ribonucleotide binding		
GO:0032555	F	purine ribonucleotide binding		
GO:0004713	F	protein tyrosine kinase activity		
GO:0000166	F	nucleotide binding		
GO:0004674	F	protein serine/threonin kinase activity		
GO:0004762	F	protein kinase activity		

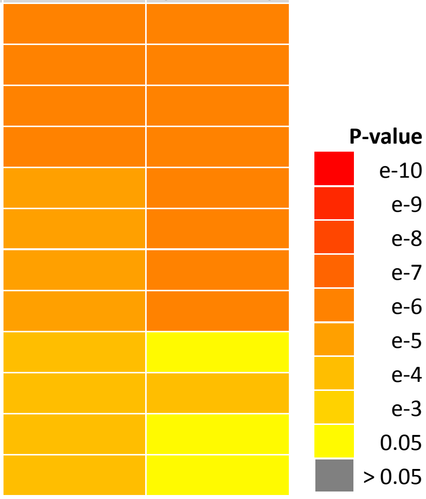

Additional file 3. Comparison of GO terms enrichment between a list of differentially expressed genes in leaves (low vs. high nitrogen, column 1) and entities in Module 6 (column 2). Only significant GO terms are displayed. Colored boxes indicate levels of statistical significance according to the scale (yellow to red represent decreasing p-values; and gray represents a non-significant result). Onto refers to the ontology category: F, Molecular function; P, Biological process; C, Cellular component.
