# Supplementary material for: Metabolic and co-expression network-based analyses associated with nitrate response in rice
Source: BMC Genomics. 2014 Dec 3;15(1):1056. doi: 10.1186/1471-2164-15-1056 (PMC4301927; doi:10.1186/1471-2164-15-1056)
Supplement: Supplementary file 5 — Additional file 5: Module Membership (MM) ranking of transcription factor related genes within clusters. (PDF 37 KB) [file 12864_2014_6767_MOESM5_ESM.pdf]

Additional file 5. Module Membership (MM) ranking of transcription factor related genes within clusters. Highest rank and p-value is compared after performing rank analysis using three different databases for rice transcription-related entities. Significant top ranked TFs are indicated in bold. n/f, none found; n/s, not significant.

Cluster	# of genes in cluster	Highest rank of TF related gene		
		**MapMan (p-value)**	**PlnTFDB (p-value)**	**DRTF (p-value)**
**Module 1**	469	24 (0.8852)	24 (0.8796)	24 (0.8062)
**Module 2**	376	40 (0.8766)	27 (0.6608)	27 (0.6624)
**Module 3**	743	27 (0.7222)	61 (0.9352)	61 (0.9089)
**Module 4**	270	10 (0.2474)	19 (0.4688)	44 (0.7022)
**Module 5**	157	5 (0.3739)	5 (0.3490)	5 (0.3217)
**Module 6**	2880	123 (0.9562)	52 (0.8822)	52 (0.7790)
**Module 7**	11861	95 (0.9578)	94 (0.9571)	108 (0.9550)
**Module 8**	694	9 (0.6725)	9 (0.5442)	9 (0.3335)
**Module 9**	1343	15 (0.5970)	15 (0.4734)	15 (0.2814)
**Module 10**	3337	7 (0.1829)	7 (0.1673)	7 (0.1340)
**Module 11**	610	**1 (0.0605)**	**1 (0.0559)**	**1 (0.0518)**
**Module 12**	390	41 (0.819)	13 (0.3029)	13 (0.2254)
**Module 13**	102	n/f	10 (0.2721)	10 (0.2193)
**Module 14**	604	**1 (0.0481)**	**1 (0.0461)**	**1 (0.0459)**
**Module 15**	8907	27 (0.9116)	27 (0.8674)	27 (0.7811)
**Module 16**	431	2 (0.0712)	8 (0.2745)	8 (0.2454)
**Module 17**	195	10 (0.4050)	14 (0.6617)	14 (0.4793)
**Module 18**	233	176 (n/s)	176 (n/s)	161 (n/s)
