# Supplementary material for: Metabolic and co-expression network-based analyses associated with nitrate response in rice
Source: BMC Genomics. 2014 Dec 3;15(1):1056. doi: 10.1186/1471-2164-15-1056 (PMC4301927; doi:10.1186/1471-2164-15-1056)
Supplement: Supplementary file 6 — Additional file 6: Summary of the number of changes in the matabolic profile in rice under different nitrate treatments according to Welch’s two sample t -test comparisons. (PDF 37 KB) [file 12864_2014_6767_MOESM6_ESM.pdf]

Additional file 6. Summary of the number of changes in the metabolic profile in rice under different nitrate treatments according to Welch's two sample t-test comparisons.

**Tissue**		**Treatment**		
		**HN / LN**	**LN-HN / LN**	**HN-LN / HN**
**Leaves**	Total number ($p<0.05$)	212	30	153
	Biochemicals (↑|↓)	109 | 103	13 | 17	106 | 47
**Roots**	Total number ($p<0.05$)	136	51	31
	Biochemicals (↑|↓)	88 | 48	5 | 46	21 | 10
