# Supplementary material for: Metabolic and co-expression network-based analyses associated with nitrate response in rice
Source: BMC Genomics. 2014 Dec 3;15(1):1056. doi: 10.1186/1471-2164-15-1056 (PMC4301927; doi:10.1186/1471-2164-15-1056)
Supplement: Supplementary file 7 — Additional file 7: Metabolic profile of amino acids in leaves and roots of rice plants subjected to different nitrate treatments. (PDF 43 KB) [file 12864_2014_6767_MOESM7_ESM.pdf]

Additional file 7. Metabolic profile of amino acids in leaves and roots of rice plants subjected to different nitrate treatments. Fold change (ratio of means) are shown. Colored cells (red=increase; green=decrease) indicate significant change for the indicated t-test ($p<0.05$).

		Leaf			Root		
**Amino acid**	**Pathway**	**HN/LN**	**LN-HN / LN**	**HN-LN / HN**	**HN/LN**	**LN-HN / LN**	**HN-LN / HN**
Alanine	Alanine and aspartate metabolism	1.16	0.75	1.42	1.42	0.92	1.08
Aspartate		6.67	1.38	0.73	2.99	1.69	1.03
Asparagine		8.08	0.88	6.92	10.29	1.27	1.28
Methionine	Cysteine, methionine, SAM, taurine metabolism	3.55	0.96	1.60	1.71	0.83	1.09
Glutamate	Glutamate metabolism	1.54	1.20	1.59	0.99	1.46	1.49
Glutamine		1.47	1.06	3.93	2.29	1.36	1.21
Glycine	Glycine, serine and threonine metabolism	1.93	0.97	2.12	1.53	1.30	1.30
Serine		1.43	0.96	2.52	1.45	0.89	1.34
Threonine		2.48	0.91	2.31	4.82	0.99	0.87
Histidine	Histidine metabolism	3.46	0.87	2.52	4.61	1.20	1.13
Lysine	Lysine metabolism	2.27	0.80	1.93	1.56	0.67	1.06
Phenylalanine	Phenylalanine and tyrosine metabolism	1.81	0.95	1.56	1.27	0.71	1.04
Tyrosine		2.56	0.99	1.95	1.31	0.78	1.01
Tryptophan	Tryptophan metabolism	0.86	0.72	1.88	0.15	0.76	9.74
Arginine	Urea cycle; arginine and proline metabolism	2.74	0.86	3.19	0.58	2.09	1.52
Proline		1.42	0.94	1.34	1.96	0.78	0.72
Isoleucine	Valine, leucine and isoleucine metabolism	3.00	0.92	2.17	2.99	0.85	0.82
Leucine		1.92	0.78	1.44	1.75	0.79	0.86
Valine		2.38	0.88	1.78	2.37	0.82	0.87
