# Supplementary material for: Metabolic and co-expression network-based analyses associated with nitrate response in rice
Source: BMC Genomics. 2014 Dec 3;15(1):1056. doi: 10.1186/1471-2164-15-1056 (PMC4301927; doi:10.1186/1471-2164-15-1056)
Supplement: Supplementary file 8 — Additional file 8: Metabolic profile of other identified compounds that presented significant changes in leaves and roots of rice plants subjected to different nitrate treatments. (PDF 52 KB) [file 12864_2014_6767_MOESM8_ESM.pdf]

**Additional file 8.** Metabolic profile of other compounds that presented significant changes in leaves and roots of rice plants subjected to different nitrate treatments. Fold change (ratio of means) are shown. Colored cells (red=increase; green=decrease) indicate significant change for the indicated t-test (p<0.05).

| | | Leaf | | | Root | | |
|---|---|---|---|---|---|---|---|
| **Compound** | **Pathway** | **HN/LN** | **LN-HN / LN** | **HN-LN / HN** | **HN/LN** | **LN-HN / LN** | **HN-LN / HN** |
| Gama amino-butyrate | Glutamate metabolism | 2.33 | 0.73 | 1.05 | 1.75 | 0.53 | 0.95 |
| Glutathione oxidized (GSSG) | Glutathione metabolism | 3.97 | 2.38 | 1.83 | 2.40 | 0.87 | 0.69 |
| 5-oxoproline | | 1.55 | 0.80 | 1.97 | 0.61 | 1.40 | 1.71 |
| 2-aminoadipate | Lysine metabolism | 2.81 | 1.09 | 2.91 | 1.43 | 1.07 | 1.07 |
| N6-acetyllysine | | 1.24 | 0.65 | 1.17 | 1.73 | 0.68 | 0.73 |
| Quinate | Phenylalanine and tyrosine metabolism | 4.24 | 1.27 | 1.02 | 2.60 | 0.84 | 0.70 |
| Putrescine | Polyamine metabolism | 1.21 | 0.96 | 2.72 | 1.66 | 0.53 | 1.17 |
| 1-kestose | Fructose, mannose, galactose, starch and sucrose metabolism | 1.73 | 0.93 | 0.65 | 1.22 | 0.91 | 0.74 |
| erythritol | | 1.52 | 0.89 | 1.70 | 1.11 | 1.00 | 1.06 |
| Fructose | | 0.89 | 0.83 | 0.84 | 1.54 | 0.71 | 0.97 |
| Galactose | | 1.76 | 0.79 | 0.91 | 1.15 | 0.68 | 1.04 |
| Raffinose | | 1.00 | 1.43 | 1.35 | 0.95 | 0.78 | 0.62 |
| Sucrose | | 2.23 | 1.40 | 0.94 | 0.21 | 0.86 | 0.63 |
| Trehalose | | 2.76 | 1.03 | 0.78 | 1.30 | 0.81 | 0.70 |
| Fructose-6-P | Glycolysis, gluconeogenesis, pyruvate metabolism | 1.82 | 0.91 | 0.88 | 1.71 | 1.43 | 1.86 |
| Glucose | | 0.93 | 0.89 | 0.97 | 1.02 | 1.45 | 1.35 |
| Glucose-6-P | | 1.88 | 0.94 | 0.83 | 1.35 | 1.77 | 1.77 |
| Glycerate | | 2.01 | 1.07 | 0.72 | 1.04 | 1.52 | 1.48 |
| Pyruvate | | 2.21 | 0.80 | 0.70 | 0.78 | 0.84 | 0.81 |
| Gluconate | Nucleotide sugars, pentose metabolism | 2.84 | 0.72 | 0.77 | 2.17 | 2.63 | 2.79 |
| Ribose | | 1.12 | 0.75 | 1.25 | 1.74 | 1.99 | 1.97 |
| Ribulose | | 2.99 | 0.62 | 0.63 | 0.93 | 1.71 | 1.25 |
| Xylose | | 1.10 | 0.92 | 0.86 | 2.05 | 3.11 | 2.41 |
| Threonate | Ascorbate and aldarate metabolism | 0.53 | 0.75 | 1.75 | 1.79 | 2.43 | 2.76 |
| Nicotinamide ribonucleotide (NMN) | Nicotinate and nicotinamide metabolism | 0.95 | 2.03 | 4.41 | 1.00 | 1.00 | 1.00 |

**Additional file 8.** (continued)

| | | | | | | | |
|---|---|---|---|---|---|---|---|
| Cis-aconitate | Krebs cycle | 4.59 | 1.12 | 2.34 | 2.19 | 0.94 | 1.09 |
| Citrate | | 1.62 | 1.19 | 1.57 | 1.94 | 0.82 | 1.26 |
| Isocitrate | | 0.53 | 0.90 | 2.64 | 1.86 | 0.76 | 0.97 |
| Malate | | 1.37 | 1.00 | 1.37 | 3.43 | 0.81 | 1.08 |
| succinate | | 0.72 | 0.93 | 1.73 | 1.44 | 1.10 | 0.88 |
| Glycerol | Glycerolipid metabolism | 1.53 | 1.01 | 1.13 | 1.45 | 0.95 | 1.08 |
| Glycerol-3-P | | 1.63 | 1.05 | 0.95 | 2.55 | 0.97 | 1.01 |
| Phosphoethanolamine | | 1.33 | 0.96 | 0.85 | 2.14 | 0.55 | 0.86 |
| Myo-inositol | Inositol metabolism | 3.72 | 1.08 | 0.80 | 1.09 | 0.72 | 1.02 |
| 2’,3’-cUMP | Pyrimidine metabolism | 0.48 | 0.58 | 1.47 | 2.09 | 0.99 | 1.09 |
| Alanylglycine | Dipeptide | 7.69 | 1.02 | 1.23 | 0.72 | 0.58 | 1.92 |
| Alanylisoleucine | | 2.29 | 0.82 | 0.80 | 1.30 | 0.90 | 1.31 |
| Alanylleucine | | 2.01 | 0.76 | 0.94 | 1.00 | 0.64 | 1.18 |
| Alanylvaline | | 2.82 | 1.07 | 0.81 | 1.32 | 0.87 | 1.06 |
| Glycylleucine | | 2.52 | 0.81 | 0.67 | 1.00 | 0.69 | 1.09 |
| γ-Glutamylglutamine | | 2.61 | 1.29 | 5.54 | 1.58 | 1.14 | 1.12 |
| Apigenin | Flavonoid | 6.98 | 0.96 | 5.84 | 1.00 | 1.00 | 1.00 |
| Quercetin-3-galactoside | | 0.20 | 1.20 | 1.76 | 1.00 | 1.00 | 1.00 |
| Luteolin-7-o-glucoside | | 0.51 | 1.08 | 1.47 | 1.00 | 1.00 | 1.00 |
| Ferulate | Phenylpropanoid | 1.64 | 0.97 | 0.90 | 0.46 | 0.73 | 1.11 |
