# Supplementary material for: Metabolic and co-expression network-based analyses associated with nitrate response in rice
Source: BMC Genomics. 2014 Dec 3;15(1):1056. doi: 10.1186/1471-2164-15-1056 (PMC4301927; doi:10.1186/1471-2164-15-1056)
Supplement: Supplementary file 9 — Additional file 9: Weighted adjacency matrix that describes pair wise similarities between probe pairs. (PDF 11 KB) [file 12864_2014_6767_MOESM9_ESM.pdf]

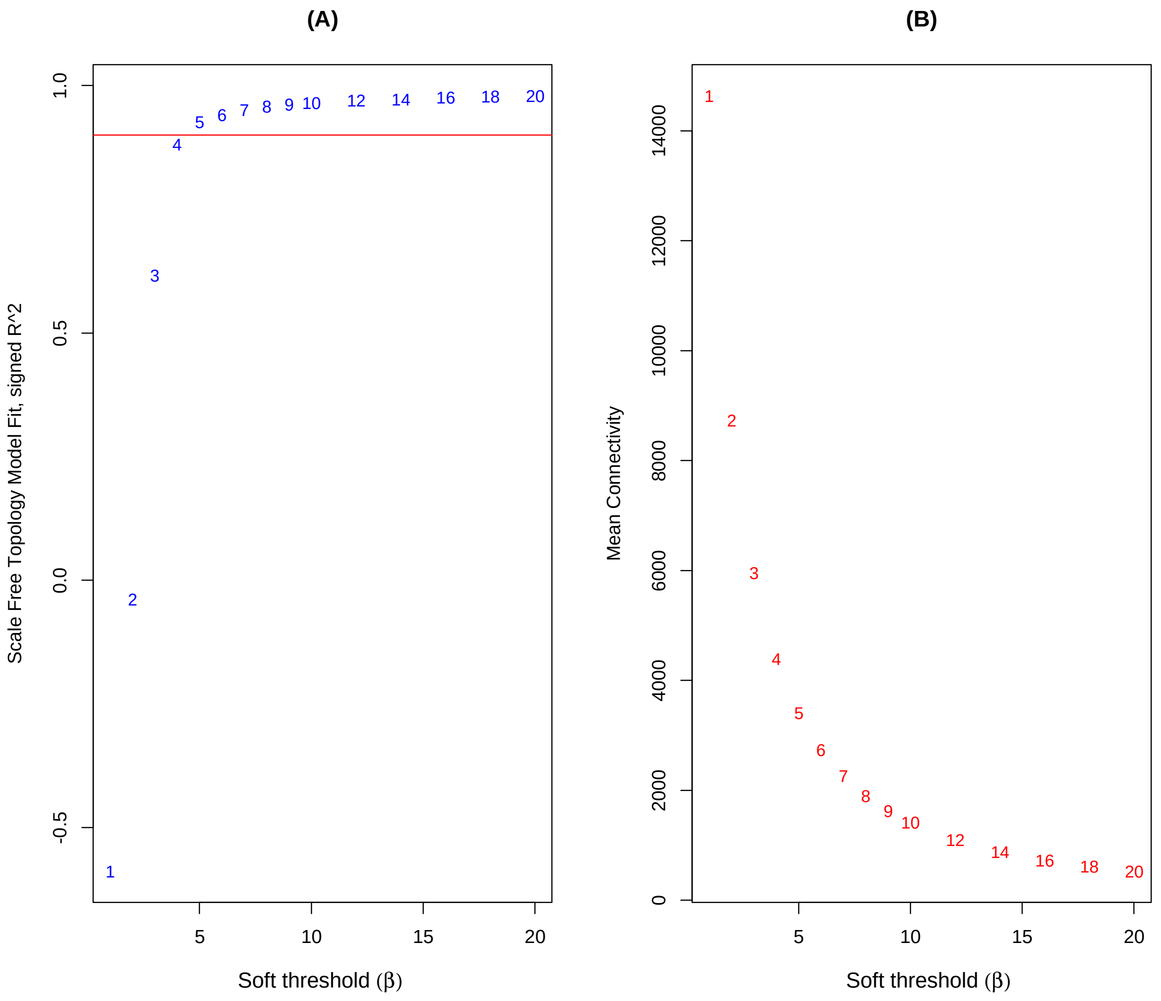

Additional file 9. Weighted adjacency matrix that describes pair wise similarities between probe pairs. (A) Scale independence of soft-threshold β. The plot describes the relationship between possible β options and the scale free topology model fit R^2 value. A soft threshold β = 4 was ultimately chosen as it was the lowest power observed before the scale free topology curve reached R^2=0.90. (B) Mean connectivity of soft-threshold β. Mean network connectivity represents the connections between all nodes. The soft threshold β = 4 maintained a high mean network connectivity and demonstrated scale-free topology.
